# Profiling Cannabinoid Contents and Expression Levels of Corresponding Biosynthetic Genes in Commercial *Cannabis* (*Cannabis sativa* L.) Cultivars

**DOI:** 10.3390/plants11223088

**Published:** 2022-11-14

**Authors:** Ae Lim Kim, Young Jae Yun, Hyong Woo Choi, Chang-Hee Hong, Hyun Joo Shim, Jeong Hwan Lee, Young-Cheon Kim

**Affiliations:** 1 Division of Life Sciences, Jeonbuk National University, 567 Baekje-daero, Deokjin-gu, Jeonju 54896, Jeollabuk-do, Korea; 2 School of Pharmacy, Jeonbuk National University, 567 Baekje-daero, Deokjin-gu, Jeonju 54896, Jeollabuk-do, Korea; 3 Department of Plant Medicals, Andong National University, 1375 Gyeongdong-ro, Andong-si 39729, Gyeongsangbuk-do, Korea; 4 LED Agri-Bio Fusion Technology Research Center, Jeonbuk National University Specialized Campus, 79 Gobong-ro, Iksan 54596, Jeollabuk-do, Korea

**Keywords:** *Cannabis*, high-performance liquid chromatography, cannabichromene, cannabidiol, tetrahydrocannabinol

## Abstract

*Cannabis* (*Cannabis sativa* L.) is widely cultivated and studied for its psychoactive and medicinal properties. As the major cannabinoids are present in acidic forms in *Cannabis* plants, non-enzymatic processes, such as decarboxylation, are crucial for their conversion to neutral active cannabinoid forms. Herein, we detected the levels of cannabidivarin (CBDV), cannabidiol (CBD), cannabichromene (CBC), and Δ9-tetrahydrocannabinol (Δ9-THC) in the leaves and vegetative shoots of five commercial *Cannabis* cultivars using a combination of relatively simple extraction, decarboxylation, and high-performance liquid chromatography analyses. The CBDV, CBC, and Δ9-THC levels were 6.3–114.9, 34.4–187.2, and 57.6–407.4 μg/g, respectively, and the CBD levels were the highest, ranging between 1.2–8.9 μg/g in leaf and vegetative shoot tissues of *Cannabis* cultivars. Additionally, correlations were observed between cannabinoid accumulation and transcription levels of genes encoding key enzymes for cannabinoid biosynthesis, including *CsCBGAS*, *CsCBDAS*, *CsCBCAS*, and *CsTHCAS*. These data suggest that the high accumulation of cannabinoids, such as CBC, Δ9-THC, and CBD, might be derived from the transcriptional regulation of *CsCBGAS* and *CsCBDAS* in *Cannabis* plants.

## 1. Introduction

*Cannabis* (*Cannabis sativa* L.), belonging to the family *Cannabaceae*, is used for various purposes such as industrial [1], ornamental [2], and pharmaceutical applications [3], and has recently attracted considerable interest as source of diverse cannabinoids, terpenes, and phenolic compounds with prominent nutraceutical potential [4]. Among them, the most well-studied pharmacologically active ingredients are cannabinoids, and in *Cannabis*, 90 cannabinoids have been identified and characterized so far [5,6]. The predominant cannabinoids, including Δ9-tetrahydrocannabinol (Δ9-THC), a psychotropic compound, and cannabidiol (CBD), with neuroprotective properties, are well-studied secondary metabolites produced in *Cannabis* plants [7,8]. *Cannabis* plants are of two types: marijuana and hemp. Marijuana may have medicinal properties and has mainly been used recreationally for the psychoactive components of Δ9-THC; hemp does not accumulate Δ9-THC to an appreciable level (<0.3%) and is valued for its medicinal compounds, fibers, and seeds, which comprise more than 25,000 known products used in industrial applications, food, supplements, textiles, paper, technical products, and personal care products [4,9,10,11]. In view of the recent global economy and research, hemp production has expanded to at least 47 countries, with Canada, China, Chile, France, and North Korea being the major producers of hemp [4]. The USA is the largest importer of hemp seeds and fibers from Canada and China [11,12,13].

**Figure 1:**
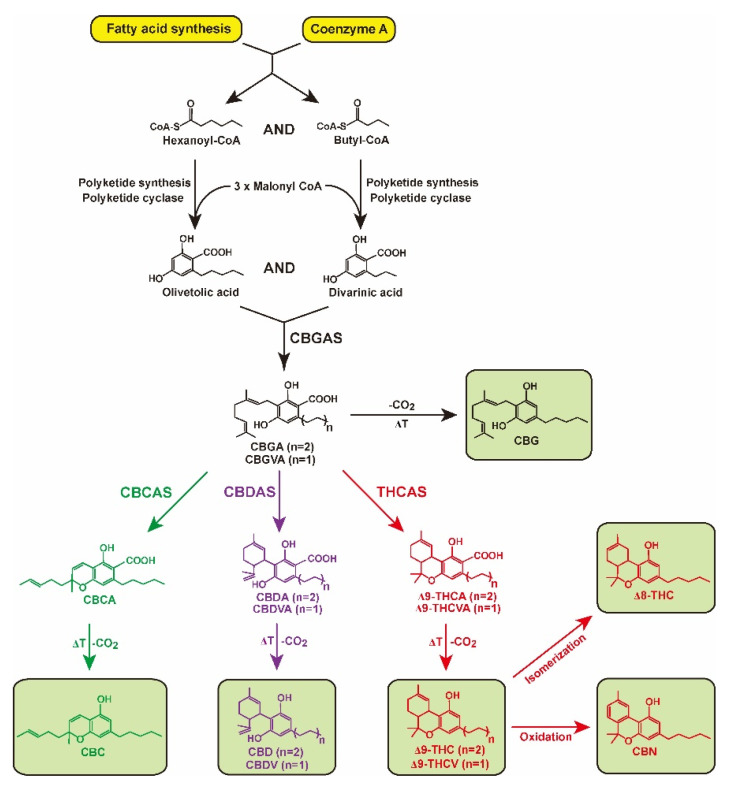
Biosynthesis pathway of cannabinoids. Hexanoyl-CoA and butyl-CoA are used as substrates to generate olivetolic and divarinic acid. Through the action of cannabigerolic acid synthase (CBGAS), olivetolic and divarinic acids are converted into cannabigerolic acid (CBGA) and cannabigerovarinic acid (CBGVA), the central precursor for cannabichromenic acid (CBCA), cannabidiolic acid (CBDA), cannabidivarinic acid (CBDVA), Δ9-tetrahydrocannabinolic acid (Δ9-THCA), and Δ9-tetrahydrocannabivarinic acid (Δ9-THCVA). After exposure to heat (ΔT), CBGA, CBCA, CBDA, CBDVA, Δ9-THCA, and Δ9-THCVA decarboxylate (-CO_2_) are converted into cannabigerol (CBG), cannabichromene (CBC), cannabidiol (CBD), cannabidivarin (CBDV), Δ9-tetrahydrocannabinol (Δ9-THC), and Δ9-tertrahydrocannabivarin (Δ9-THCV), respectively. Cannabinoids Δ8-tetrahydrocannabinol (Δ8-THC) and cannabinol (CBN) are formed by isomerization and oxidation, respectively, of Δ9-THC.

The cannabinoid biosynthesis pathway in *Cannabis* plants has been well-studied and determined [14]. Cannabinoids are synthesized from fatty acids and isoprenoid precursors (Figure 1). Hexanoyl-CoA and butyl-CoA, derived from fatty acid biosynthesis, are used in the biosynthesis of olivetolic and divarinic acids using polyketide cyclase enzymes, respectively [14,15]. Prenyltransferase, also known as cannabigerolic acid synthase (CBGAS), produces cannabigerolic acid (CBGA) and cannabigerovarinic acid (CBGVA), which are derived from C21 and C19 precursors formed from olivetolic and divarinic acids, respectively [14,15]. The oxidocyclases cannabichromenic acid synthase (CBCAS), cannabidiolic acid synthase (CBDAS), and tetrahydrocannabinolic acid synthase (THCAS) promote oxidative cyclization of the monoterpene moiety, generating cannabichromenic acid (CBCA), cannabidiolic acid (CBDA), cannabidivarinic acid (CBDVA), Δ9-tetrahydrocannabinolic acid (Δ9-THCA), and Δ9-tetrahydrocannabivarinic acid (Δ9-THCVA), respectively [16,17,18,19]. Genetic studies on the most prominent oxidocyclases, *CBDAS*, *CBCAS*, and *THCAS* in *Cannabis* plants revealed that the three genes encoding these enzymes have a single exon, with THCAS and CBCAS sharing 92% amino acid identity with each other and having 84% and 83% amino acid sequences, respectively, identical to those of CBDAS [16,20,21,22,23].

Cannabinoids are stored in carboxylic acid forms, and neutral cannabinoids do not occur at significant levels in plants; however, acidic cannabinoids can be readily decarboxylated to their corresponding neutral forms of cannabigerol (CBG), cannabichromene (CBC), cannabidivarin (CBDV), CBD, Δ9-tertrahydrocannabivarin (Δ9-THCV), and Δ9-THC by nonenzymatic thermal decarboxylation when exposed to light, heat, or combustion [24]. The different isomers, Δ8-tetrahydrocannabinol (Δ8-THC) and cannabinol (CBN), result in isomerization of the double bond in the alicyclic carbon ring and oxidative degradation of Δ9-THC, respectively [25].

The main challenges faced by many researchers are the limited reliable data on the cannabinoid composition of various *Cannabis* strains, the production variability of these compounds within the *Cannabis* plants, the effects of the cultural environment on the quality of cannabinoids production, and the qualitative methods offering an effective way for the separation and quantification of the neutral cannabinoids. The purpose of this study was to develop a time- and cost-saving method for the quantification of important bioactive neutral cannabinoids in several *Cannabis* cultivars using a simple extraction and decarboxylation method followed by high-performance liquid chromatography (HPLC) analysis. To quantify the content of neutral cannabinoids in *Cannabis* plants, new heating decarboxylation conditions were adopted using reference mixtures of acidic and neutral cannabinoids, and the decarboxylation of acidic cannabinoids and conversion of their corresponding neutral cannabinoids were estimated. The vegetative shoots and leaves of five commercial *Cannabis* cultivars were analyzed under quantitative analytical conditions to determine the content of eight neutral cannabinoids. Additionally, the correlation between cannabinoid levels and expression patterns of their biosynthesis-related genes was determined.

## 2. Results

### 2.1. New Decarboxylation Conditions of Acidic Cannabinoids for Liquid Chromatography (LC) Analysis

Gas chromatography (GC) and LC assays are the most widely used analytical techniques to analyze cannabinoids in *Cannabis* [26,27]. Although GC coupled with mass spectrometry has been used as the official quantification and identification method [28,29], acidic cannabinoids cannot be analyzed without decarboxylation or derivation because the GC-heated injector port converts the acidic form to a neutral form by heat-derived decarboxylation. The decarboxylation of Δ9-THCA at an intermediate temperature (225 °C) was maximal but with only 65% conversion to Δ9-THC, and a significant loss in THC was observed at 300 °C [30]. This indicated that GC analysis was unsatisfactory in that particular study.

**Figure 2:**
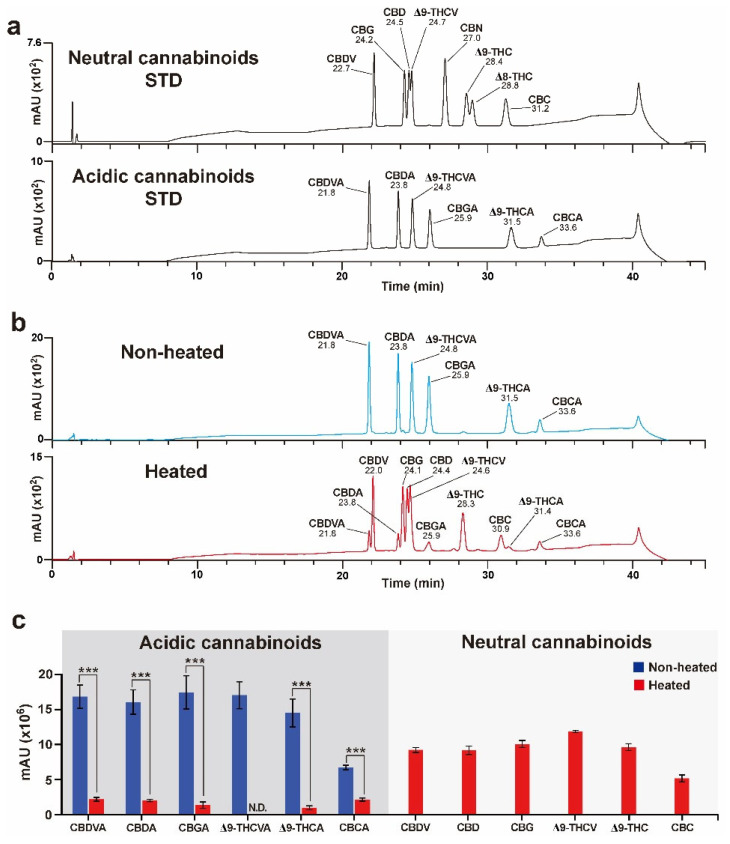
Decarboxylation of the acidic cannabinoid mixture. (**a**) High-performance liquid chromatography (HPLC) chromatograms of standard (STD) neutral and acidic cannabinoid mixtures. (**b**) Resulting HPLC chromatograms after heat and non-heat treatment of acidic cannabinoid mixtures. (**c**) Results for the decarboxylation of acidic cannabinoids. CBDVA, CBDA, CBGA, Δ9-THCVA, Δ9-THCA, and CBCA signals were decreased, and their corresponding neutral cannabinoid signals were newly detected after heat treatment. The Δ9-THCVA signal was non-detectable (nd) owing to the overlap in retention time between Δ9-THCVA and Δ9-THCV after heat treatment. Error bars indicate the SEM of three replicates (Student’s *t*-test, *** *p* < 0.001).

In the present study, LC analysis was used as an alternate chromatography technique to detect both acidic and neutral cannabinoids in *Cannabis*. To analyze as many neutral cannabinoids as possible, eight neutral cannabinoid (CBDV, CBG, CBD, THCV, CBN, Δ9-THC, Δ8-THC, and CBC) mixtures were used as reference standards (Figure 2a). These neutral cannabinoids are not predominant in *Cannabis*, and decarboxylation conditions of their corresponding acidic forms were needed prior to the assay. Additionally, the conversion efficiencies of six acidic cannabinoids (CBDVA, CBGA, CBDA, THCVA, Δ9-THCA, and CBCA) to their neutral forms under specific decarboxylation conditions were assessed. A previous study using Δ9-THCA and CBDA showed that the conversion of Δ9-THCA and CBDA to Δ9-THC and CBD, respectively, reached completion with relatively less loss after decarboxylation at 105 °C or 110 °C for 1 h [28,31]. Decarboxylation was conducted at 110 °C for 1 h, and conversion efficiencies were measured using six acidic cannabinoid mixtures as references (Figure 2b). Acidic cannabinoids, including CBDVA, CBDA, CBGA, Δ9-THCVA, Δ9-THCA, and CBCA, were considerably decreased by heat treatment, resulting in conversion to their neural cannabinoid forms, such as CBDV, CBD, CBG, Δ9-THCV, Δ9-THC, and CBC, respectively (Figure 2c).

### 2.2. Evaluation of New Decarboxylation Conditions Using Cannabis Plant Extracts

**Figure 3:**
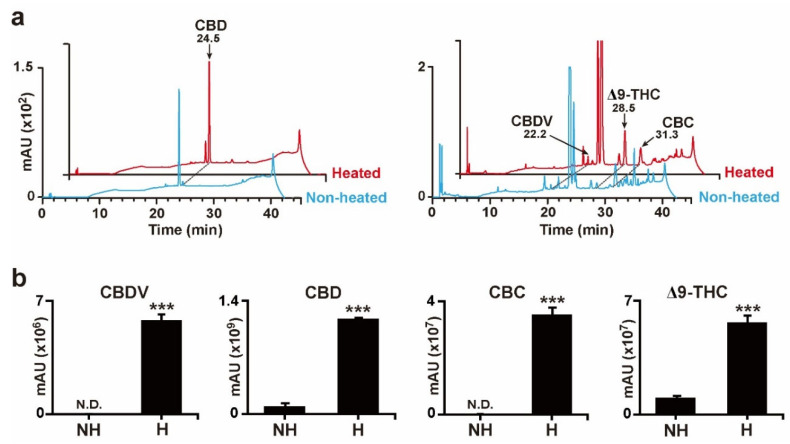
Decarboxylation results of the *Cannabis* plant extract. (**a**) HPLC chromatograms of enriched neutral cannabinoids, CBD, CBDV, Δ9-THC, and CBC after heat treatment. (**b**) Results for enriched CBD, CBDV, Δ9-THC, and CBC signals after heat treatment. nd indicates non-detectable. Error bars indicate the SEM of three replicates (Student’s *t*-test, *** *p* < 0.001).

To elucidate the effects of decarboxylation treatment on *Cannabis* plant extracts, the extracts from *Cannabis* plants were heated under the same conditions as those used for the decarboxylation of standard acidic cannabinoids (Figure 2b). The levels of CBDV, CBD, CBC, and Δ9-THC, which were almost undetectable before heating, showed significantly increased signals after heat treatment (Figure 3a,b). These results indicated that the heating decarboxylation reaction performed in the present study was appropriate for the subsequent investigation of neutral cannabinoid composition profiles in various *Cannabis* cultivars.

### 2.3. Analysis of Cannabinoid Composition and Levels in Different Tissues of Cannabis Cultivars

**Figure 4:**
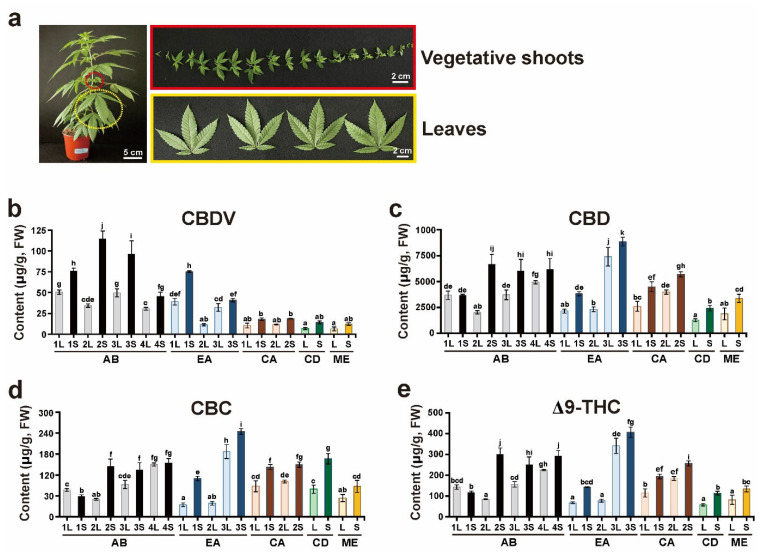
Quantitative analysis of neutral cannabinoids. (**a**) Vegetative shoots (S) and true leaves (L) of 2-month-old *Cannabis* plants were used for cannabinoid analysis. (**b**) CBDV, (**c**) CBD, (**d**) CBC, and (**e**) Δ9-THC levels were quantified from S and L extracts of three individuals each of abacus 2.0 (AB) and early abacus (EA), two individuals each of cherry abacus (CA), and a single plant each of cherry diesel (CD) and merlot (ME). The Y axis represents the contents of cannabinoids in fresh weight (FW). Different lowercase letters indicate significant difference (*p* < 0.05) according to Tukey’s test.

Considerable literature describes the sites of cannabinoid biosynthesis in *Cannabis* plants [32,33,34,35]. Cannabinoids and terpenes accumulate in the glandular trichomes of the plants; in particular, female flowers show the highest density of glandular trichomes. Moreover, to maintain proper inbred lines, female plants are cultivated exclusively to prevent fertilization with pollen from other male flower plants because *Cannabis* is an anemophilous plant that is pollinated by wind [36,37]. However, considerable time and space were needed to maintain the various *Cannabis* cultivars separately for harvesting whole buds of mature female flowers. In addition, continuous artificial selection through extensive breeding between local landraces and commercial cultivars leads to allelic heterogeneity in *Cannabis* cultivars, thereby resulting in the production of individual plants showing different phenotypes, although the same *Cannabis* cultivars have been cultivated [38]. Therefore, the present study was designed to use two tissues at the early vegetative stage of *Cannabis* to profile the composition of neutral cannabinoids in independent individual plants of five *Cannabis* cultivars (Figure S1). Vegetative shoots and leaves at the 2-month-old stage after germination were harvested from individual *Cannabis* plants of each cultivar, abacus 2.0 (AB), early abacus (EA), and cherry abacus (CA) as well as single plants of cherry diesel (CD) and merlot (ME) cultivars (Figure 4a).

Four cannabinoids, CBDV, CBD, CBC, and Δ9-THC, were detected in 11 individual *Cannabis* plants germinated from seeds of the five cultivars (Table S1). Among the detected cannabinoids, CBDV content was the lowest, and CBDV concentrations in the vegetative shoots of the AB cultivar showed high amounts (Figure 4b). The concentrations of CBD were the highest in most cultivars in comparison to those of other cannabinoids (Figure 4c). The leaves and vegetative shoots of EA3 showed the highest content of CBD, but the CBD levels of EA1 and 2 were relatively low. Both CBC and Δ9-THC showed similar patterns and levels, and the highest amounts were detected in the leaves and vegetative shoots of EA3 (Figure 4d,e). The levels of CBDV, CBD, CBC, and Δ9-THC in the vegetative shoots were higher than those in the leaves of all cultivars analyzed in this study. The levels of the four cannabinoids were significantly different in individual plants of the same cultivars, AB and EA.

**Figure 5:**
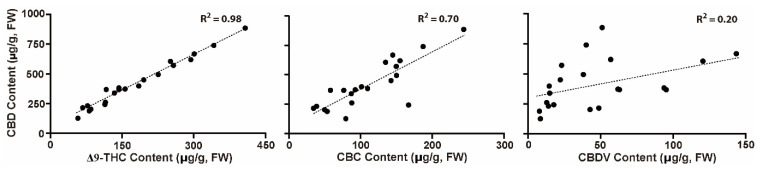
Paired comparison of cannabinoid levels. Comparison of CBD content (Y axis) with the contents of Δ9-THC, CBD, and CBDV (X axis) detected in vegetative shoots and leaves of independent individuals of the *Cannabis* cultivars AB, EA, CA, CD, and ME. Cannabinoid values in each sample are plotted.

The levels of CBDV, CBD, CBC, and Δ9-THC detected in the 11 individual *Cannabis* plants were plotted to determine correlations between them (Figure 5). The CBD levels ranged between 1.2–8.9 μg/g, whereas for Δ9-THC, CBC, and CBDV, the ranges were 0.05–0.41 μg/g, 0.067–0.24 μg/g, and 0.006–0.12 μg/g, respectively (Table S1). There were significant positive and slight correlations between CBD and Δ9-THC, CBD and CBC, and CBD and CBDV levels, with R^2^ values of 0.98, 0.70, and 0.20, respectively. This result suggests that some cultivars prone to biosynthesizing high CBD in their vegetative shoots or leaves may result in increased levels of the biosynthesized Δ9-THC, CBC, and CBDV.

### 2.4. Expression Profiling of CsCBGAS, CsCBCAS, CsCBDAS, and CsTHCAS in Cannabis Cultivars

**Figure 6:**
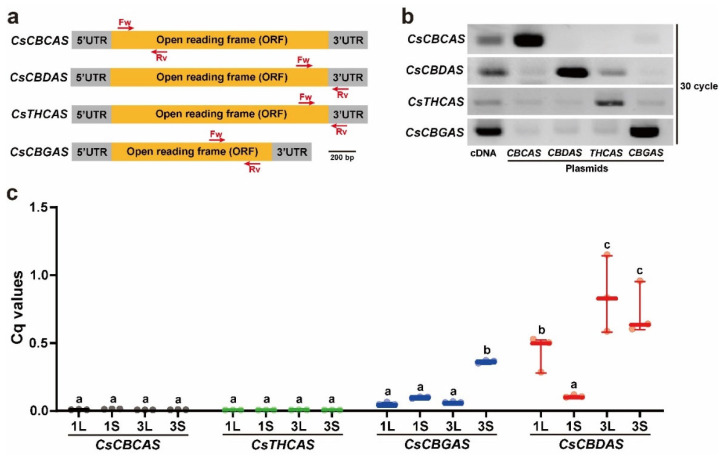
Specificity of primers for cannabinoid biosynthesis genes and RT-qPCR assay results. (**a**) Diagram of the primer sets designed for RT-qPCR assay of *CsCBCAS*, *CsCBDAS*, *CsTHCAS*, and *CsCBGAS*. Locations of gene-specific primers used for the RT-qPCR experiment are indicated. (**b**) Specificity of the designed primer sets for *CsCBCAS*, *CsCBDAS*, *CsTHCAS*, and *CsCBGAS*. The expected amplicon bands of *CsCBCAS*, *CsCBDAS*, *CsTHCAS*, and *CsCBGAS* were mainly obtained from cDNA and plasmids containing the corresponding open reading frames (ORFs) or 3′-untranslated regions (UTRs). (**c**) Transcriptional levels of *CsCBCAS*, *CsCBDAS*, *CsTHCAS*, and *CsCBGAS* in leaves (L) and vegetative shoots (S) of EA1 and EA3 plants. Different lowercase letters indicate significant difference (*p* < 0.05) according to Tukey’s test.

To determine the relationship between cannabinoid accumulation and expression of cannabinoid biosynthesis genes, the transcription levels of four cannabinoid biosynthesis genes, *CsCBGAS*, *CsCBCAS*, *CsCBDAS*, and *CsTHCAS*, were analyzed using real-time quantitative (RT-qPCR) analysis. The expression levels were determined in two individual plants, EA1 and EA3, which showed representatively high or low amounts of CBD, CBC, and Δ9-THC (Figure 4 and Table S1). Given the high similarity of the target genes at the nucleotide level, specific primer sets were designed and validated for their specificity (Figure 6a). We first performed RT-PCR and PCR analyses using different templates, including cDNA synthesized from the leaves of *Cannabis* plants and plasmids containing the open reading frames (ORFs) of *CsCBCAS* and *CsCBGAS* or additional 3′-untranslated regions of *CsCBDAS* and *CsTHCAS* as positive controls (Figure 6b). The results showed that our designed primer sets specifically amplified the corresponding target genes, although faint bands were detected in the PCR reactions using the *CsCBDAS* and *CsCBGAS* primer sets. Next, we conducted RT-qPCR analysis to examine the expression levels of *CsCBGAS*, *CsCBCAS*, *CsCBDAS*, and *CsTHCAS* in the leaves and vegetative shoots of two individual plants (EA1 and EA3) (Figure 6c). The transcript levels of *CsCBCAS* and *CsTHCAS* were lower than those of *CsCBGAS* and *CsCBDAS* in all samples. The highest transcript amounts of *CsCBGAS*, an enzyme that produces precursors for CBC, Δ9-THC, and CBD, were found in EA3S. The expression levels of *CsCBDAS* were the highest in the leaves and vegetative shoots of EA3, wherein the highest accumulation of CBD was detected. These results suggested that the high accumulation of cannabinoids, such as CBC, Δ9-THC, and CBD, may be derived from transcriptional regulation of two cannabinoid biosynthetic genes, *CsCBGAS* and *CsCBDAS*, in *Cannabis* plants.

## 3. Discussion

The most abundant cannabinoid compounds among C21 terpeno-phenolics were Δ9-THCA and CBDA, followed by CBGA and CBCA. Their associated C19 homologs were CBGVA, Δ9-THCVA, and CBDVA (Figure 1) [39]. Four oxidocyclases, CBGAS, CBCAS, CBDAS, and Δ9-THCAS, convert CBGA to CBCA, CBDA, and Δ9-THCA, respectively [16,17]. These acidic cannabinoids are nonenzymatically converted to their corresponding neutral forms, which also occur minimally in *Cannabis* plants [24]. Although GC and HPLC analytical techniques were applied to determine acidic and neutral cannabinoid concentrations, GC analysis systems, which comprise a heated injection port prior to column separation, are not optimal for acidic forms, as heating results in the conversion of acidic cannabinoids to neutral forms [40]. However, the HPLC system can analyze both acidic and neutral cannabinoids because the process does not involve high temperatures. In the present study, we applied an HPLC analytical system to evaluate the decarboxylation efficiencies of acidic cannabinoids and detect neutral cannabinoids. In previous studies on the decarboxylation of acidic cannabinoids, only three acidic cannabinoids, Δ9-THCA, CBDA, and CBGA, were focused on, and the conversion of their corresponding neutral forms under a range of temperatures and times were confirmed [28,31]. Our study provided additional information about the decarboxylation of acidic cannabinoids, CBDVA, Δ9-THCVA, and CBCA, by analyzing the products of their neutral forms. Most acidic cannabinoids were highly decarboxylated, but the efficiency of the formed corresponding neutral forms was relatively lower than that before decarboxylation (Figure 2c). This might be due to compound loss during the heat treatment; the decrease in the total concentration of acidic cannabinoids (Δ9-THCA, CBDA, and CBGA) and their neutral forms (Δ9-THC, CBD, and CBG) upon decarboxylation at high (110 °C) and low (60 °C) temperatures was reported in previous studies [28,31].

Although novel extraction procedures, such as ultrasound-assisted, microwave-assisted, pressurized liquid, and supercritical fluid extraction, have been applied in the past decade for good recovery of targeted cannabinoid compounds, each technique requires an appropriate solvent type, temperature, pressure, and treatment time [41,42]. Analytical methods, such as simple color tests, thin-layer chromatography, GC, GC–mass spectrometry, LC, LC–mass spectrometry, and their combinations are generally sufficient for the identification of cannabinoids according to the purpose of the analysis [29]. However, the maintenance costs of analytical instruments and cannabinoid management policies in different countries should be considered when selecting analytical methods. In the present study, a combination of relatively simple extraction procedures, decarboxylation procedures, and instruments compared to those used in previous studies [41,42] was applied for the analysis of various neutral cannabinoid compositions in commercially available *Cannabis* cultivars (Figure 2 and Figure 3). This simple combination could be applicable for the primary screening of medicinal *Cannabis* cultivars containing targeted cannabinoids. Additionally, optimized extraction and decarboxylation conditions for high solubility, good recovery, and bioactive stability of the desired cannabinoid might be needed.

Four cannabinoids, CBDV, CBD, CBC, and Δ9-THC, were detectable in vegetative shoots and leaves in 11 individual plants of five *Cannabis* cultivars (Figure 4 and Table S1). Vegetative shoots showed higher levels of cannabinoids than leaves. These results are similar to those of previous studies showing higher levels of cannabinoids in the leaves of the upper nodes [43,44]. The accumulation patterns of the four cannabinoids differed in the same cultivar lines. In particular, the differences in CBDV, CBD, CBC, and Δ9-THC concentrations among the EA cultivar plants (EA1, EA2, and EA3) were the largest. These findings were unexpected and suggest that there is a high possibility of genetic variation, even within seed lots produced from the same cultivar. This notion is supported by reports that continuous artificial selection through extensive breeding between local landraces and commercial cultivars leads to allelic heterogeneity in *Cannabis* cultivars [38]. A previous study revealed that the transcription levels of *CsTHCAS* and *CsCBDAS* reflect the chemical phenotype of *Cannabis* plants [45]. Additionally, single nucleotide polymorphisms of *CBCAS* and divergent sequences of *CsTHCAS* and *CsCBDAS* were identified in a genotype investigation using 13 *Cannabis* cultivars [45].

## 4. Materials and methods

### 4.1. Plant Materials

Abacus 2.0 (AB), early abacus (EA), cherry abacus (CA), cherry diesel (CD), and merlot (ME) *Cannabis* (*C. sativa* L.) seeds were kindly provided by the LED Agri-Bio Fusion Technology Research Center (Jeonbuk National University Specialized Campus, Iksan, Korea). *Cannabis* seeds were grown in a growth room at 25 °C under long-day conditions (photoperiod, 16 h:8 h, light: dark) at a light intensity of 120 μmol m^−2^ s^−1^. True leaves and other tissues of 2-months-old *Cannabis* plants were used for cannabinoid analysis.

### 4.2. RNA Expression Analyses

Sequences of *CsCBGAS*, *CsCBCAS*, *CsCBDAS*, and *CsTHCAS* were obtained from the *Cannabis* genome browser (http://genome.ccbr.utoronto.ca/, accessed on 12 October 2020). Primer sequences used in the present study are listed in Table 1. To examine the expression levels of *CsCBGAS*, *CsCBCAS*, *CsCBDAS*, and *CsTHCAS* in *Cannabis* plants, RT-qPCR analysis was conducted. Total RNA was extracted from *Cannabis* leaves using the RiboEX Total RNA kit (GeneAll, Seoul, Korea). RNA quality was determined using a Nanodrop ND-200 spectrophotometer (Nanodrop Technologies, Wilmington, DE, USA), and only high-quality RNA (A260/A230 > 2.0, and A260/A280 > 1.8) was used for subsequent experiments. Synthesis of cDNA was performed using 5 μg of the total RNA using the ReverTra Ace qPCR RT Master Mix kit (Toyobo, Osaka, Japan) following the manufacturer’s protocol, and RT-qPCR analysis was conducted in 96-well plates using a CFX real-time system (Bio-Rad, Sanra Clara, CA, USA) and a THUNDERBIRD SYBR qPCR mix (Toyobo). In accordance with the best-established RT-qPCR practices, *CsEF1a* (*JP452083*) is one of the most stable reference genes in *Cannabis* [46]. Three independent biological replicates were analyzed. The oligonucleotide sequences used for RNA expression analysis are provided in Table 1. To determine the relative abundance of the transcripts, the data were analyzed using the Bio-Rad CFX Manager software (Bio-Rad).

### 4.3. Plasmid Constructions

The full-length ORFs for *CsCBGAS*, *CsCBCAS*, *CsCBDAS*, and *CsTHCAS* were amplified from the total RNA of the *Cannabis* plants using RT-PCR with gene-specific primer sets (Table 1). The ORFs of the genes were cloned into TA vectors following the protocol in the RBC cloning vector kit (RBC Bioscience, New Taipei City, Taiwan), and the produced plasmids were used for the validation of primer specificity in the RT-qPCR assay.

### 4.4. Extract Preparation and Decarboxylation Reactions

The collected *Cannabis* samples were ground to a powder in a mortar with liquid nitrogen. Samples (0.15 g) were homogenized in 1 mL of 100% acetonitrile, sonicated for 1 h, and incubated at 55 °C for 1 h. The supernatants were recovered by centrifugation at 13,000× *g* for 10 min and then filtered through a 0.45 μm polyvinylidene difluoride membrane (Pall, NY, USA). The extracts were concentrated in a speed vacuum concentrator (Eyela, Tokyo, Japan) for 1 day. Decarboxylation reactions were performed as previously described [31]. For decarboxylation reactions, the individual samples were placed in an oven for 1 h at 110 °C. The decarboxylated extracts were reconstituted in 120 μL acetonitrile prior to injection into the HPLC system.

### 4.5. Analytical Conditions for HPLC of Cannabinoids

The HPLC system comprised an LC-20AD pump, SPD-20A UV/VIS detector, CBM-20A communications bus module, and an SIL-20AC autosampler (Shimadzu, Kyoto, Japan) combined with a C_18_ 3.9 × 300 mm μ-Bondapak column (Waters, MA, USA). Separation using HPLC was conducted using two mobile phases: A phase containing 0.1% formic acid in water and B phase containing 0.1% formic acid in acetonitrile. The sample was injected at a total volume of 10 μL, and the flow rate of the solvent was maintained at 2 mL/min. The sample was separated using the following gradient elution profile: 25% B for 5 min, increased to 50% B and maintained for 10–15 min, increased to 70% B and maintained for 20–30 min, increased to 100% B, maintained for 33–37 min, and subsequently restored to 25% B and maintained for 10–45 min. Ultraviolet signals were monitored at a fixed wavelength of 225 nm. Acidic (six components: CBCA, CBDA, CBDVA, CBGA, Δ9-THCA, and Δ9-THCVA) and neutral (eight components: CBC, CBD, CBDV, CBG, CBN, Δ8-THC, Δ9-THC, and THCV) cannabinoid mixtures were purchased from Supelco (Sigma-Aldrich, St. Louis, MO, USA).

## 5. Conclusions

This project was undertaken to quantify the important bioactive neutral cannabinoids in *Cannabis* plants by HPLC analysis and investigate the correlations between expressions of cannabinoid synthases genes and their corresponding cannabinoids’ accumulation. Simple extraction procedures, decarboxylation procedures, and instruments were applied for the analysis of various neutral cannabinoid compositions in commercial *Cannabis* cultivars, and specific primers were designed for univocal identification and transcriptional analysis of *CsCBCAS*, *CsCBDAS*, *CsTHCAS*, and *CsCBGAS*. This study has shown that there is a possibility of genetic variation among the same cultivars, and the high accumulation of cannabinoids may be derived from transcriptional regulation of cannabinoid biosynthetic genes in *Cannabis* plants.

Further studies with a focus on the polymorphism analysis of cannabinoid biosynthesis-related genes, including *CsCBGAS*, *CsCBCAS*, *CsCBDAS*, and *CsTHCAS*, in diverse *Cannabis* cultivars containing different major cannabinoids, including CBDV, CBD, CBC, and Δ9-THC, are therefore suggested to reveal relationships between the biosynthesized cannabinoid levels and genetic variations. Finally, given the preferred *Cannabis* plant sources for specific medical cannabinoids produced in a limited period of time, the application of the clustered regulatory interspaced short palindromic repeats (CRISPR)/CRISPR-associated protein (Cas) system, a widely adopted genome engineering platform, might be preferable for developing new *Cannabis* cultivars that enhance or inhibit the biosynthesis of desirable cannabinoids.

## Figures and Tables

**Table 1:** Information of primers used in this study.

Gene Name (ID)	Purpose	Sequence (5′ to 3′)	Direction
*CsCBCAS* (KJ469378)	Gene cloning	ATGAATTGCTCAACATTCTCCTTTTGG	F
		TTAATGATGATGCGGTGGAAGAGG	R
	RT-qPCR	TAACAATCCAGCAAATCCAAAATTCA	F
		GGAGCAGAGAATACTGGCC	R
*CsCBDAS* (KP970860)	Gene cloning	ATGAAGTGCTCAACATTCTCCTTTTG	F
		TTAATGACGATGCCGTGGAAG	R
	RT-qPCR	AAGTGAAAACCCTGGTTGATCC	F
		GAGCATACACAGTACATCCGG	R
*CsTHCAS* (KJ469378)	Gene cloning	ATGAATTGCTCAGCATTTTCCTTTTGG	F
		TTAATGATGATGCGGTGGAAGAGG	R
	RT-qPCR	TTAGGAAAAACTAATCATGCG	F
		CAATAAATGTATGTATGGTATAATGATTA	R
*CsCBGAS* (BK010648)	Gene cloning	ATGGAGGTCTCATCAGTTTGC	F
		TTATATAAATACATATACAAAG	R
	RT-qPCR	CCATATCGAGTCATTTGGGCTT	F
		GGCAAAAGCAATAGTCATACCC	R
*CsEF1a* (JP452083)	RT-qPCR	GCTTTGATACCCCTCCACAA	F
		AGTATCCGCGAGCTTGACAT	R

## Data Availability

The data presented in this study are available upon request from the corresponding author.

## Supplementary Materials

The following supporting information can be downloaded at: https://www.mdpi.com/article/10.3390/plants11223088/s1, Figure S1: Two-month-old *Cannabis* plants used in this study. Table S1: The content levels of CBDV, CBD, CBC, and Δ9-THC, cannabinoids detected in prepared in this study.
