# Supplementary material for: Profiling Cannabinoid Contents and Expression Levels of Corresponding Biosynthetic Genes in Commercial Cannabis (Cannabis sativa L.) Cultivars
Source: Plants (Basel). 2022 Nov 14;11(22):3088. doi: 10.3390/plants11223088 (PMC9697443; doi:10.3390/plants11223088)
Supplement: Supplementary file 1 [file plants-11-03088-s001.zip › plants-2023058-supplementary.pdf]

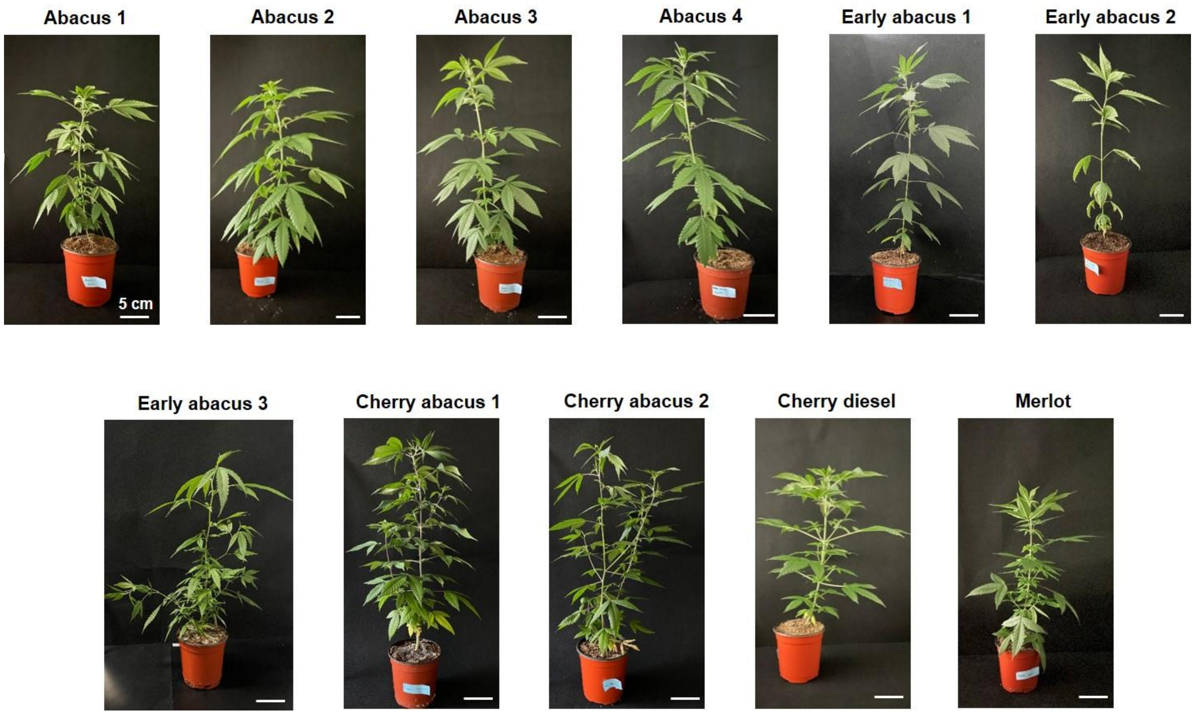


**Figure S1.** Two months-old *Cannabis* cultivars used in this study. Four independent individuals of abacus 2.0 (AB), three independent individuals of early abacus (EA), two independent individuals of cherry abacus (CA), and single plant of cherry diesel (CD) and merlot (ME). The numbers indicate independent individual plant germinated from same seeds.

**Table S1.** The contents of four cannabinoids in eleven individual plants of five *Cannabis* cultivars.

| Plant | Organ | Cannabinoids (μg/g, FW[a]) | | | |
|---|---|---|---|---|---|
| | | CBDV | CBD | CBC | Δ9-THC |
| Abacus 1 | L | 50.4 ± 2.7 | 3676.3 ± 403.2 | 76.7 ± 4.1 | 143.3 ± 10.5 |
| | S | 76.3 ± 3.1 | 3675.4 ± 109.8 | 58.1 ± 4.8 | 116.8 ± 7.6 |
| Abacus 2 | L | 34.3 ± 2.1 | 2024.5 ± 151.3 | 49.9 ± 2.8 | 84.8 ± 2.4 |
| | S | 114.9 ± 9.1 | 6673.3 ± 949.6 | 145.0 ± 20.3 | 300.8 ± 31.7 |
| Abacus 3 | L | 49.6 ± 4.8 | 3718.8 ± 464.1 | 92.5 ± 11.6 | 156.3 ± 13.4 |
| | S | 96.5 ± 15.8 | 6053.9 ± 1088.6 | 134.9 ± 20.5 | 251.1 ± 37.5 |
| Abacus 4 | L | 30.5 ± 1.7 | 4940.3 ± 162.4 | 150.1 ± 4.4 | 225.6 ± 3.9 |
| | S | 45.6 ± 4.9 | 6188.7 ± 1046.3 | 155.1 ± 12.4 | 293.7 ± 25.7 |
| Early abacus 1 | L | 39.1 ± 3.9 | 2143.2 ± 190.2 | 34.4 ± 5.5 | 67.6 ± 4.2 |
| | S | 75.1 ± 1.0 | 3823.1 ± 187.3 | 109.9 ± 6.3 | 143.4 ± 2.0 |
| Early abacus 2 | L | 11.3 ± 1.4 | 2314.1 ± 225.9 | 38.7 ± 5.5 | 77.7 ± 6.7 |
| Early abacus 3 | L | 32.1 ± 4.7 | 7398.1 ± 894.9 | 187.2 ± 19.9 | 341.7 ± 36.6 |
| | S | 40.9 ± 2.1 | 8851.6 ± 421.4 | 243.9 ± 8.4 | 407.4 ± 24.7 |
| Cherry abacus 1 | L | 10.3 ± 2.7 | 2607.2 ± 478.9 | 87.8 ± 15.8 | 115.2 ± 20.0 |
| | S | 17.8 ± 1.6 | 4496.1 ± 480.8 | 142.5 ± 7.5 | 195.1 ± 11.2 |
| Cherry abacus 2 | L | 11.7 ± 0.5 | 3976.5 ± 201.1 | 101.3 ± 4.3 | 184.9 ± 9.6 |
| | S | 18.5 ± 0.4 | 5707.5 ± 252.0 | 149.8 ± 7.4 | 257.4 ± 12.2 |
| Cherry diesel | L | 6.8 ± 1.4 | 1260.7 ± 147.2 | 79.5 ± 11.7 | 57.6 ± 5.3 |
| | S | 14.2 ± 1.5 | 2422.5 ± 266.9 | 166.9 ± 15.2 | 114.1 ± 8.8 |
| Merlot | L | 6.3 ± 2.3 | 1882.1 ± 563.5 | 53.5 ± 10.1 | 81.2 ± 22.3 |
| | S | 12.0 ± 1.6 | 3385.7 ± 395.7 | 87.1 ± 17.1 | 134.0 ± 14.4 |

Concentration expressed as μg/g (avg ± std dev): [a]Fresh weight, FW.
